# Depression Can Affect Anyone: Report on Three Waves of National Representative Survey in Poland Measured With PHQ-8

**DOI:** 10.1155/2024/2241722

**Published:** 2024-10-17

**Authors:** Piotr Toczyski, Michał Feliksiak

**Affiliations:** ^1^Maria Grzegorzewska University, Warsaw, Poland; ^2^Public Opinion Research Center, Warsaw, Poland

## Abstract

We conducted three surveys on representative random samples of adult Polish residents drawn from the citizens' register. They were conducted in June 2022 (*N* = 1050), October 2022 (*N* = 1041), and February 2023 (*N* = 982). Interviews were conducted using a mixed-mode technique (CAPI, CATI, and CAWI). Our key findings are that at least mild symptoms of depression are exhibited by a quarter of adults surveyed (25.8%), including a tenth (9.4%) with moderate or more severe symptoms. Translated to the population, that is more than 7.5 million Poles with at least mild symptoms and more than 2.7 million with more severe symptoms, respectively. The scale of depression symptoms is characterized by a certain seasonality. Fewer people experience them in spring than in autumn and winter. The most significant risk factors include, first of all, a poor economic situation, the presence of other health problems, and young age. There is also a higher risk for women and residents of large cities.

## 1. Introduction: PHQ-9, Its Shorter Version PHQ-8 and National Sample Studies

For two decades, the Patient Health Questionnaire-9 (PHQ-9) is proven to be a widely used screening tool for depression in clinical and research settings. From dozens of articles related to the PHQ-9 and its use in various populations and settings, some may be mentioned to show the broadness of geographic, medical, and social contexts. One study by AlHadi et al. [1] translated and validated the PHQ-9 into Arabic, finding it to be a valid and reliable tool for measuring depression in a Saudi Arabian sample. Another study by Arroll et al. [2] validated the PHQ-2 and PHQ-9 as effective tools for screening for major depression in a primary care setting, with both scales showing high levels of accuracy and sensitivity. Several studies have examined the effectiveness of psychological interventions for depression in various populations. Baumeister, Hutter, and Bengel [3] reviewed the literature on psychological and pharmacological interventions for depression in patients with diabetes mellitus and found that psychological interventions, including cognitive behavioral therapy, were effective in treating depression in this population as measured by PHQ-9. Other studies have examined the factor structure of the PHQ-9 and its relationship to other measures. Huang et al. [4] found that the PHQ-9 had a two-factor structure, with one factor measuring cognitive and affective symptoms and the other factor measuring somatic symptoms. Founders of PHQ screeners Kroenke, Spitzer, and Williams [5] found that the PHQ-9 had good construct and criterion validity and was highly correlated with other measures of depression. Studies have also examined the use of the PHQ-9 in specific populations, such as cancer patients [6] and patients with coronary heart disease [7]. These studies found the PHQ-9 to be a valid and reliable tool for measuring depression in these populations. In summary, the PHQ-9 is a widely used screening tool for depression with good validity and reliability in various populations and settings.

The use of the PHQ-8 in social survey research with national samples was noted. Studies demonstrate the utility of the PHQ-8 as a measure of depression in social survey research with national samples. The findings from a recent study, possibly the most extensive to date examining the internal framework, dependability, and comparability across different nations of a self-reported mental health evaluation tool, indicate that the PHQ-8 demonstrates satisfactory reliability and equivalence across the 27 European countries examined. These outcomes underscore the viability of comparing PHQ-8 scores within Europe and could offer valuable insights for enhancing the screening and evaluation of depressive symptoms' severity at a European level [8].

Depression is a common mental health condition that can have significant negative impacts on an individual's well-being and quality of life. Thus, the PHQ-8 and PHQ-9, but even their shorter versions such as PHQ-2, are commonly used screening tools for depression in primary care settings. However, they have also been utilized in social survey research and national samples to assess the prevalence of depression in broader populations. Several studies have demonstrated the utility of the PHQ-8 and PHQ-9 in social survey research and national samples. For example, a study by Levis, Benedetti, and Thombs [9] found that both the PHQ-8 and PHQ-9 had good psychometric properties and were valid and reliable measures of depression in large, diverse national samples. In another analysis of more than 50 studies, PHQ-8 and PHQ-9 total scores were similar, with sensitivity minimally reduced with the PHQ-8, but similar specificity [10].

In conclusion, the PHQ-8 and PHQ-9 are valid and reliable measures for screening depression in various settings, including social survey research, national samples, and primary care settings in many countries from different continents, leading us to its potential inclusion also in the context of Poland, the Central and Eastern European country with the mental health culture merging legacies of stigma, undertreatment, and westernized modernity expressed in European Union (EU) accession in 2004. Depression screening including PHQ-9 in primary healthcare has been recommended in Poland 15 years later, within the framework of EU-funded guidelines [11].

The current section has been focused on PHQ approach to depression screening, leading us to focus on Poland's specificity. The question remains how to access the sample truly representing the population, when focusing on Polish adult population. In Poland, selected population studies may be conducted with the use of PESEL. So called PESEL stands for Powszechny Elektroniczny System Ewidencji Ludności (Universal Electronic System for Registration of the Population) and has been the official identification number utilized in Poland since 1979. Consisting of 11 digits, it serves as a unique identifier for individuals and remains unchangeable. Mandatory for both permanent residents and temporary residents residing in Poland for more than 2 months, the PESEL number is essential for official identification purposes. Its benefit is that the PESEL number contains date of birth of its holder, which contributes to the quality assurance of the research activities performed with its use.

Accessing PESEL register is possible only for official administrative use or for selected research purposes. Approaching the sample of Poles accessed via PESEL register with PHQ-8 has been considered a significant research task worth undertaking to better understand how PHQ screening approach works in the population.

The current research aims to address several specific gaps. Firstly, PHQ-8 studies are typically conducted infrequently. In this instance, the approach involves conducting ongoing PHQ-8 tracking of depression symptoms in a sample of similar respondents (recruited in the same manner, though not identical individuals) to help fill the knowledge gap regarding changes over time and the seasonality of symptoms. Secondly, the social structure, as reflected in the sociodemographic questionnaire, will provide insights into the distribution of depressive symptoms across different social groups, based on typical sociodemographic criteria. Thirdly, the research aims to bridge the gap in knowledge regarding potential referrals among the surveyed adult population (aged 18–75). This information may be informative for social and health policy not only in Poland but also in similar countries. Replicating the results could make a valuable contribution to other healthcare systems and mental health and psychosocial support interventions, including community-based interventions.

## 2. Methodology

We conducted three PHQ-8 surveys on representative random samples of adult Polish residents drawn from the PESEL register. The survey was conducted using a mixed-mode procedure on a representative named sample of adult Polish residents drawn from the PESEL register. Each respondent independently selected one of the methods: face-to-face interview with an interviewer (CAPI method); telephone interview after contacting an interviewer (CATI)—the respondent's contact information was received in an announcement letter; and self-completion of an online survey, which was accessed based on the login and password provided to the respondent in the announcement letter. In all three cases, the survey had the same set of questions and structure.

They were conducted in 2022: in June (30.05–9.06; *N* = 1050) and October (3–13.10; *N* = 1041) and, in 2023, in February (6–19.02; *N* = 982). Interviews were conducted using a mixed-mode technique, meaning that respondents could choose a convenient way to answer the questionnaire. These included face-to-face (CAPI) or telephone (CATI) interviews with an interviewer, as well as self-completion of the survey via the Internet (CAWI).

In each edition of the survey, respondents were asked the same set of eight questions, which are a shortened version (PHQ-8) of the Brief Patient Health Questionnaire, Depression Module (PHQ-9), based on criteria for measuring depression from the Diagnostic and Statistical Manual of Mental Disorders, Fourth Edition (DSM-IV). The respondents were not asked about any other illnesses, including the ones that may affect the development of depressive disorders.

The research is nonmedical, so no written informed consent was obtained from participants. Its equivalent has been the freedom to participate in research after receiving the announcement letter. The total number of participants does not add up to 3073 in some tables due to the respondents' decision of nonresponding to questions focused, e.g., on political views, use of Internet, and participation in religious activities.

How the survey results can be extrapolated to Poland? Describing the sampling methodology will make readers aware of the statistical methods by which the results are generalizable. The research encompasses the adult population of residents registered within Poland. Interviews are conducted with a group of approximately 1000 respondents. According to publicly available information from the center for public opinion research (Dz. Ustaw [12]), the sample for the study is selected in a multistage random sampling process. There are four stages which contribute to the process:

Stage 1: The first stage involves selecting the sampling frame. The sampling frame is a register containing information about all individuals belonging to the surveyed population. For research purposes, the PESEL register is utilized, containing data on all individuals registered in Poland.
Stage 2: The second stage of sampling involves stratifying the population into layers or subpopulations. If the sample is to be representative, it must be drawn from the entire population. Therefore, respondents are selected from all strata. The stratification serves to gain greater control over the territorial dispersion of the sample. The surveyed population is divided based on categories of residential areas. Six categories are distinguished: rural areas; towns with populations up to 19,999; towns with populations from 20,000 to 49,999; towns with populations from 50,000 to 99,999; towns with populations from 100,000 to 499,999; and cities with populations exceeding 500,000 (Cracow, Łódź, Poznań, Warsaw, and Wrocław). Not every province's population is divided into all six subgroups. Ultimately, the research center receives approximately 80 strata.
Stage 3: The next step is sample allocation. The research center decides how many respondents to draw from each stratum. Allocation is prepared to obtain a sample in which each stratum is represented proportionally to its size.
Stage 4: The final stage of sampling is random selection. It occurs in two stages. The first involves randomly selecting municipalities within each stratum. The number of selected municipalities is proportional to the size of the stratum, and municipalities are chosen with probabilities proportional to their population size. If a stratum consists of only one municipality, the first stage is skipped. In the next step, exactly 10 individuals (or multiples of this number if the municipality was selected multiple times) belonging to the surveyed adult population of Polish citizens are randomly selected from each municipality. This method results in named samples, consisting of precisely specified individuals.

The more detailed methodological information can be accessed via regular public communication of the abovementioned public research center. The procedure is well established in Polish social research landscape.

## 3. Results

### 3.1. Symptoms of Depression: A Combined Approach

To what extent were the respondents affected by particular mental states and behaviors that could indicate depression? In the 2 weeks preceding the survey, more than half of the respondents (52.9%) felt fatigue or lack of energy for at least a few days; nearly two-fifths (38.5%) had trouble sleeping, intermittent sleep, or too much sleep; and one-third (32.2%) felt sad, depressed, or hopeless. In the 14 days prior to the survey, more than a quarter of respondents (28.2%) experienced little interest in the activities they were doing on at least a few occasions, a fifth (19.4%) had trouble focusing, and a similar number (18.2%) were dissatisfied with themselves, feeling that they were up to no good or that they were letting themselves or loved ones down. In the 2 weeks preceding the measurement, about one-sixth of adults (15.5%) experienced a lack of appetite or overeating for at least a few days. Roughly one-tenth (11.4%) were bothered during this time by slowness of speech or movement or irritability resulting in excessive mobility.

Over the 2 weeks preceding the survey, about one-fifth of adults experienced feelings of fatigue or lack of energy (19.1%) and trouble sleeping (18.2%) on more than half of the days. About a tenth experienced a lack of interest in their activities for more than half of the days or more often (10.7%), feelings of sadness, depression, or hopelessness (10.6%). Slightly less severe were such conditions as deficits in attention and concentration (6.5%), feelings of dissatisfaction with oneself (6.3%), lack of appetite or overeating (6.1%), and slowing or agitation regarding speech or movements (4.5%).

Counting respondents' answers to the above eight questions created a synthetic measure of depressive symptoms. The index is based on the sum of answers to eight questions on a 4-point scale, on which 1 means that the condition was not experienced at all and 4 means that it was experienced almost every day. The index takes values from 8 to 32. Values from 8 to 12 indicate the absence of depressive symptoms; from 13 to 17, mild symptoms; from 18 to 22, moderate symptoms; from 23 to 27, moderately severe symptoms; and from 28 to 32, severe symptoms.

**Figure 1:**
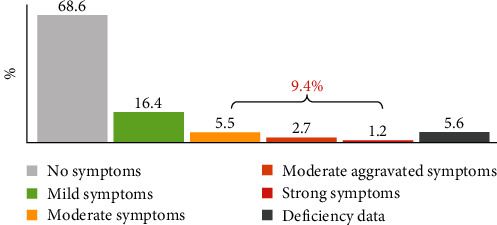
Synthetic index of depressive symptoms.

Based on it, it can be said that mild depressive symptoms are presented by one-sixth of adults (16.4%) and more severe (i.e., at least moderate) by less than one-tenth (9.4%), with one-twentieth (5.5%) presenting moderate symptoms, three in a hundred (2.7%) presenting moderately severe symptoms, and one in a hundred (1.2%) presenting severe symptoms. More than two-thirds of adults (68.6%) show no symptoms of depression. This is illustrated in Figure 1.

Translating these results to the adult population of Poland (37.8 million people), it can be said that at least mild symptoms of depression affect more than 7.5 million people, of which more than 2.7 million have more severe symptoms, i.e., moderate, more than 1.6 million; moderately severe, more than 790,000; and severe, about 350,000 people.  Table 1 is the reference point for these numbers.

### 3.2. Seasonality

The questions were asked three times: in late spring, autumn, and winter. Do the results obtained differ in any way depending on the season? In general, most of the mental states and behaviors addressed by the questions are more often evident in autumn and winter than in spring. If the June measurement is taken as a reference point, the biggest differences are in sleep problems (up 5.5 points in October and 8.7 points in February), loss of interest (5.9 points and 7.6 points, respectively), and feelings of fatigue or lack of energy (5.4 points and 7.1 points, respectively).

It is also worth looking at seasonal differences in terms of frequent (i.e., more than half of the days) occurrence of individual symptoms. In such a view, the biggest differences between the spring and autumn/winter seasons are marked in sleep problems (relative to the June measurement, an increase of 5.7 points in October and 4.9 points in February), appetite disorders (up 4.5 points and 4.7 points, respectively), and—although here we register mostly differences between June and October—experiencing feelings of fatigue and lack of energy (up 6.3 points and 1.9 points, respectively), sadness and depression (up 4.6 points and 2.1 points, respectively), and loss of interest (up 3.6 points and 2.2 points, respectively). In the case of symptoms such as feelings of fatigue and lack of energy, as well as sadness and despondency, it is possible to speak to some extent of an intensification in autumn and a weakening in winter. Symptoms such as attention deficit, feelings of dissatisfaction with oneself, and slowing down or agitation regarding speech or movements do not seem to be subject to seasonality.

**Figure 2:**
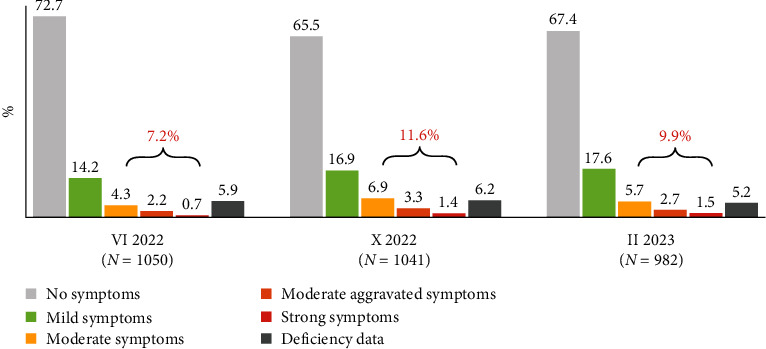
Symptoms of depression—three measurements.

A compilation of the values of the synthetic index of depressive symptoms from the three measurements confirms the existence of seasonality of depressive symptoms, i.e., an increase in the percentage of adults who experience them, in autumn, and the relative persistence of this scale in winter. At least mild depressive symptoms in the 14 days preceding the survey were 21.4% in June, 28.5% in October, and 27.5% in February. Considering the scale of at least moderate symptoms is, respectively, 7.2%, 11.6%, and 9.9%.  Figure 2 is an illustration of these numbers.

### 3.3. Sociodemographic Variations of Depression Scale

The occurrence of depressive symptoms in the 2 weeks preceding the survey is most strongly associated with a subjectively poor assessment of one's own material situation. At least mild symptoms were experienced during this period by more than half of respondents dissatisfied with their own material situation (50.3% vs. 25.8% among all respondents) and more severe (i.e., at least moderate) symptoms by a quarter of them (24.0% vs. 9.4%). The dependence of mental health on economic factors is also reflected, albeit to a lesser extent, by income received. Mild or more severe symptoms of depression are more common among respondents from households with per capita incomes of less than PLN 1500 (at least mild, 34.1%, and at least moderate, 13.5%).

A factor that clearly co-occurs with depressive symptoms is young age and—largely related to this—being a member of the student group. Among those aged 18–24, at least mild symptoms of depression are presented by more than a third (35.7%) and more severe by 16.4%. Among those who are studying or learning, it is two-fifths (39.9%) and one-fifth (20.1%), respectively.

Other health problems also appear to be important in the determinants of depression. Although the survey did not include questions in which respondents could assess their health or indicate what they suffer from, they could count themselves among the group of pensioners in which at least mild symptoms of depression are manifested by nearly two-fifths (38.9%) and more serious ones by less than one-fifth (17.4%). It should be borne in mind that in the case of pensioners, health problems, in addition to the fact that they can affect the deterioration of social contacts, lower mobility, and other related phenomena, translate into a worse material situation. Among pensioners, more than twice as many as among the general population rate their material situation as bad (18.9% vs. 7.4%).

The category that stands out from the others with more frequent mild depressive symptoms are those who are economically inactive, taking care of the home, or not working for reasons other than pension, retirement, or study (34.2%). These people are not significantly worse off than the general population. It seems that in this case, a factor affecting mental health may be the lack of professional activity, which is also generally associated with interpersonal contacts and some social activity.

At least mild symptoms of depression stand out for residents of large (100,000 to 499,999 population) and largest (half-million and larger) cities. They affect about a third of them (for large cities, 31.1%, and the largest, 33.8%).

It is also worth noting the role of worldview and lifestyle factors. Respondents who identify with the left and are not involved in religious practices stand out from the rest with more frequent symptoms of depression. Among those with leftist views, about a third of respondents (32.0%) have at least mild symptoms of it, and 13.5% have more severe symptoms. Among those who do not participate in religious practices, the figures are 31.1% and 13.9%, respectively. However, it should be borne in mind that this relationship may to some extent be a function of age. The youngest respondents (18–24) are more likely to identify with the left (32.0% vs. 21.9%) and more likely not to be religiously involved (37.8% vs. 21.6%) than the general population.

Some differences can be observed in the extent of depression according to Internet use or nonuse. Mild or more severe symptoms are more common among those who are offline than among Internet users (31.0% vs. 24.1%).

There is also a disparity in the extent of depression between men and women. Mild or more severe symptoms of depression affect women more than men (29.7% vs. 21.1%). It should be borne in mind, however, that the disparity is not necessarily due to women's greater incidence of depression than men's but—perhaps more importantly—to the different degree to which they admit to their particular symptoms. When it comes to the more serious, or at least moderate, symptoms of depression, there is no significant difference by the gender of respondents.  Table 2 reflects the abovementioned information and integrates them in the concise form which can be informative for further possible strategies.

Analyzing the symptom scale while controlling for the gender and age of respondents, it can be seen that young age increases the risk of depressive symptoms in both men and women. Women under the age of 25 stand out the most in terms of both mild and more severe symptoms. More than two-fifths of them (43.8%) show at least mild symptoms of depression, and more than one-fifth (22.7%) show moderate or more severe symptoms. The oldest group of women, those 65 and older (35.5%), also emerges as slightly more at risk for depression in this regard.  Table 3 illustrates the abovementioned phenomena.

Controlling for gender, it can be observed that while the size of the locality in which the respondents live is related to the risk of depression for men (the larger the locality, the higher the percentage of people with mild or more severe depressive symptoms), this does not play such a significant role among women. For more information about place of residence, gender and depression symptoms Table 4 are the reference point.

A material situation perceived subjectively as bad increases the risk of depression for both men and women.  Table 5 is an illustration of the abovementioned assessment of own material conditions in the context of depression.

As for the worldview characteristics of respondents by gender expressed in Table 6, it can be noted that left-wing beliefs co-occur with a slightly higher than average incidence of experiencing depressive symptoms in both men and women. In contrast, as expressed in Table 7, the lack of religious commitment noticeably increases the risk of experiencing depressive symptoms for women. In the case of men, there is less such significance.

From logistic regression analysis, in which the dependent variable is the presence of at least moderate depressive symptoms (or lack thereof) and the independent variables, gender, age, place of residence, and assessment of one's own financial situation and pensioner status, it shows that the risk of depression is statistically significantly increased by poor financial situation (with respect to average/good odds ratio of 3.485), age 18–24 (with respect to older respondents, the odds quotient is 2.395), being a pensioner (odds quotient = 2.194), being a woman (odds quotient = 1.660), and living in a large city (100,000 and larger relative to smaller towns, odds quotient = 1.534). If the same model is applied separately to both genders, for men, poor financial situation (odds ratio = 3.629), being on a pension (odds ratio = 2.421), living in a large city (odds ratio = 1.890), and young age (odds ratio = 1.877) are statistically significant. For women, the hierarchy of determinants is slightly different. Poor material situation also ranks first (odds ratio = 3.400), but this is followed—unlike among men—by young age (odds ratio = 2.846), being on a pension (odds ratio = 1.995), and living in a large city (odds ratio = 1.355).

Apart from the abovementioned tables and figures, there are appendix tables following the text. Table A1 provides essential data on PHQ-8 depression index, whereas Tables A2, A3, A4, A5, A6, A7, A8, and A9 reflect PHQ-8 eight symptoms referred to sociodemographic variables. They have no direct reference in the text; however, they are valuable addition for interested stakeholders who would like to focus their research or policy work on the depression symptoms.

## 4. Discussion

The PHQ-8 and PHQ-9 are commonly used measures for screening depression in primary care settings. A study by Kokoszka, Jastrzębska, and Obrębski [13] evaluated the psychometric properties of the PHQ-9 in primary care settings in Poland, finding that it had good internal consistency, test–retest reliability, and criterion validity. This study suggests that the PHQ-9 is a useful tool for assessing depression in primary care in Poland. The PHQ-8 could be a useful alternative to the longer PHQ-9 in settings where time and resources are limited. These measures can help identify patients who may benefit from further evaluation or treatment for depression. Clinicians in Poland can use either of these measures to efficiently and effectively screen for depression in primary care settings.

With our current three waves, the clinicians in Poland and beyond may be better prepared to understand the symptoms in general population and refer them better for their clinical practice. The methodology of the current study uses CAPI, CAWI, and CATI which is a powerful methodological tool, inclusive for all adult age groups. The young adult group (18–24) may be distinguished, with higher prevalence of depressive symptoms than the general populations. From other studies, we know that depression may coexist with other common illnesses related with other age groups, such as migraine, which may also be reflected in the guideline-oriented treatment of depression in national healthcare systems contexts. Based on the Polish large cohort study, depression was found in more than 21% of respondents [14]. This result is in line with the PHQ-8 screening performed in the current study; the details of both data sets may need to be compared in a yet separate future study. However, even at the current stage, the results may apply to highlighting the need of combined screening and clinical procedures, including PHQ-9 and following its results to introduce further clinical procedures to focus on comorbidities. According to the first epidemiological surveys assessing the mental health of Poles, EZOP I and EZOP II (2012 and 2020) based on WHO methodology (ICD 10; DSM 4 and 5), depressive disorder affects less than 4% of Polish population [15]. From the German public health context, we know that the overall agreement between both measures, PHQ-9 and CIDI, was moderate [16], which opens field of debate about the gap between initial screening and clinical diagnosis of depression as measured by both tools.

Nevertheless, the PHQ-based approach has been recognized in official guidelines of the General Chamber of Physicians and Polish Psychiatric Society in Poland as good screening methods in primary healthcare [11]. The information about prevalence of potential depressive symptoms in the population of Poland may be thus informative for healthcare policy and management, enabling smooth referral.

Since 2021, the pan-European multilanguage noncommercial iFightDepression.eu website, cofunded by the European Commission's Third Health Program, enables self-testing of Poles using PHQ-9. Given the seasonality of depressive symptoms as measured with PHQ-8, one can imagine the website becoming part of national healthcare referral system, in accordance with the abovementioned guidelines for primary healthcare and family medicine. Self-testing and bringing the PHQ-9 screening result by patient may save time in general practitioner's office, leading to more relevant treatment. PHQ-2, PHQ-8, or PHQ-9 screening is not equivalent of clinical diagnosis, but it is relevant for Polish healthcare integrating with the emerging European Health Union, constituted also by the EU new comprehensive approach to mental health.

Given the prevalence of PHQ-measured depressive symptoms in the population of Poland, the health policy may be eager to apply large-scale and low intensiveness interventions such as World Health Organization's Self Help + (SH+) or Problem Management + (PM+) booklets, which contain modules referring to self-management (but in no case self-treatment) of mild-to-moderate depressive symptoms. The WHO mhGAP intervention recently introduced to Poland may also be a good policy solution, activating primary healthcare in response to mental health symptomatology prevalence in the population.

Digital mental health tools addressing depression symptomatology, in addition to treatment as usual, already exist in the Germany, the Poland's Western neighbor. According to a recent study, the most frequently recommended e-mental health interventions by specialist doctors were deprexis (currently the only one covered by health insurance), whereas moodgym was most often recommended by GPs and iFightDepression by clinicians and psychotherapists [17]. One can imagine Polish healthcare system to follow similar route and introduce digital tools, integrating them with referrals system. The first one to date has been clinically tested abovementioned EU-funded intervention iFightDepression, guided tool based on cognitive behavioral principles and PHQ-9 monitoring over time [18]. Introducing such tools on a mass scale, leading to better psychoeducation in society, can be a policy response to the challenge of mass depression symptoms. Controlling sociodemographic variables during such intervention, both in public communication and clinical settings, seems to be a prerequisite for relevance of the intervention.

Identifying and referring individuals to care using PHQ-9 or its shorter versions may also require community-based approach, such as alliances against depression mentioned by Linskens et al. [19] as the most promising multistrategy intervention among those involving, inter alia, community screening for depression using leaflets. Such approach has also been recently introduced in Poland with the local awareness raising of public mental health in the capital city of Poland through launch of local alliance against depression [20]. Given the relatively big percent of declared symptoms, PHQ health questionnaires could be widely distributed on the leaflets but also on the posters or other out-of-home media, during such future interventions in Poland. It would certainly support the referral system to proper care, recently advanced in Poland with building over 100 of the community mental health centers which attempt at community-level, deinstitutionalized interventions for public mental health.

The current study approach to PHQ-based self-testing and screening, expressed in the set of interrelated tables with the study results, may lead policy analysts to build strategic recommendations toward depression, based on sociodemographic criteria and also variables less usual in public healthcare approach, such as position in class structure, political orientation, or religious practices. It will also be informative for European-level health and social policy, as mental health is the emerging topic to be addressed across policies.

## Figures and Tables

**Table 1:** Total number of adults in Poland described by synthetic index of depressive symptoms.

Synthetic index of depressive symptoms	Total number of adults	
No symptoms	20,156,372	—
Mild symptoms	4,806,199	—
Moderate symptoms	1,606,429	2,747,813
Moderately severe symptoms	791,112	
Severe symptoms	350,272	
Data gaps	1,651,457	—

**Table 2:** Synthetic index of depression symptoms in Polish population (*N* = 3073).

	At least mild symptoms	At least moderate symptoms	Number of people
	%	%	
Total	25.8	9.4	3073
Gender			
Men	21.1	7.3	1446
Women	29.7	11.1	1627
Age (years)			
18–24	35.7	16.4	292
25–34	25.5	9.5	485
35–44	22.0	7.5	599
45–54	21.1	7.2	491
55–64	22.1	7.7	479
65 years and older	30.4	10.5	727
Place of residence			
Village	22.1	8.4	1262
City to 19,999	26.5	10.1	399
20,000–99,999	24.8	7.5	672
100,000–499,999	31.1	12	442
500,000 or more residents	33.8	12.4	299
Education			
Elementary/middle school	28.4	12.8	467
Basic vocational	24.7	9.1	712
Medium	26.1	9.6	1093
Higher	24,6	7.3	800
Social and professional group			
Staff of managers, especially with higher education	22.1	8.6	367
Medium staff, technicians	26.0	8.3	148
Admin office employees	26.9	8.0	219
Service workers	23.9	7.8	255
Skilled workers	13.5	5.4	351
Unskilled laborers	23.3	12.7	119
Farmers	18.3	3.7	134
Self-employed	14.6	3.6	132
Unemployed	28.6	11.7	52
Retirees	29.1	9.8	798
Pensioners	38.9	17.4	127
Pupils and students	39.9	20.1	154
Homemakers and others	34.2	11.4	217
S/he works at:			
Institute of state, public	22.0	8.3	400
A partnership of private owners and the state	17.0	6.6	357
Private sector, nonfarm	23.4	8.7	843
Private farming	19.4	3.6	140
Per capita income			
Up to PLN 1499	34.1	13.5	489
From PLN 1500 to PLN 999	28.4	12.7	418
From PLN 2000 to PLN 2999	25.3	7.6	557
From PLN 3000 to PLN 3999	29.9	11.1	302
PLN 4000 and above	24.9	8.2	320
Hard to say	22.0	8.7	412
Refusal to answer	18.0	5.3	575
Evaluation of own material situation			
Wrong	50.3	24.0	227
Medium	30.5	11.3	1258
Good	18.5	5.8	1588
Participation in religious practices			
Several times a week	29.3	13.1	147
Once a week	22.2	6.8	1120
1–2 times a month	24.0	8.6	396
Several times a year	28.5	9.4	679
Does not participate at all	31.1	13.9	648
Political views			
Left	32.0	13.5	647
Center	24.7	7.8	800
Right	23.7	7.2	1046
Hard to say	25.8	11.8	463
Using the Internet			
Yes	24.1	8.3	2349
Not	31.0	12.5	704

**Table 3:** Synthetic index of depression symptoms in Polish population, age distribution (*N* = 3073).

Age	At least mild symptoms		At least moderate symptoms	
	Men (%)	Women (%)	Men (%)	Women (%)
18–24 years	29.3	43.8	11.0	22.7
25–34	20.8	30.1	6.9	12.1
35–44	18.6	24.5	7.6	7.5
45–54	15.9	25.5	4.3	9.7
55–64	19.4	24.5	6.8	8.6
65 years and older	24.1	35.5	8.2	12.5
Total	21.1	29.7	7.3	11.1

**Table 4:** Synthetic index of depression symptoms in Polish population, place of residence distribution (*N* = 3073).

Place of residence	At least mild symptoms		At least moderate symptoms	
	Men (%)	Women (%)	Men (%)	Women (%)
Village	16.4	27.6	6.3	10.4
City to 19,999	20.8	31.2	7.9	12.2
20,000–99,999	21.2	27.9	5.2	9.6
100,000–499,999	27.8	34.2	9.1	14.6
500,000 or more inhabitants	32.4	34.6	13.2	11.7
Total	21.1	29.7	7.3	11.1

**Table 5:** Synthetic index of depression symptoms in Polish population, self-assessed material conditions distribution (*N* = 3073).

Assessment of own material conditions	At least mild symptoms		At least moderate symptoms	
	Men (%)	Women (%)	Men (%)	Women (%)
Bad	45.9	53.9	20.2	27.3
Medium	25.2	34.6	9.1	13.0
Good	15.2	21.8	4.4	7.0
Total	21.1	29.7	7.3	11.1

**Table 6:** Synthetic index of depression symptoms in Polish population, political view distribution (*N* = 3073).

Political views	At least mild symptoms		At least moderate symptoms	
	Men (%)	Women (%)	Men (%)	Women (%)
Left	26.8	35.7	10.8	15.3
Center	19.9	28.6	6.6	8.8
Right	20.9	27.5	5.4	9.7
Total	21.1	29.7	7.3	11.1

**Table 7:** Synthetic index of depression symptoms in Polish population, participation in religious practice distribution (*N* = 3073).

Participation in religious practices	At least mild symptoms		At least moderate symptoms	
	Men (%)	Women (%)	Men (%)	Women (%)
Several times a week	22.2	31.5	11.1	14.3
Once a week	17.9	25.7	4.3	8.8
1–2 times a month	19.7	27.7	8.2	8.9
Several times a year	24.6	32.7	8.0	10.9
Does not participate at all	24.0	39.1	10.4	17.9
Total	21.1	29.7	7.3	11.1

**Table 8:** Synthetic index of depression symptoms in Polish population, distribution (*N* = 3073).

Sociodemographic variables	Depression index (recorded)						Number of people
	Lack of symptoms	Mild symptoms	Moderate symptoms	Moderately severe symptoms	Severe symptoms	Refusal to answer	
	%	%	%	%	%	%	
Total	68.6	16.4	5.5	2.7	1.2	5.6	3073
Gender							
Men	73.4	13.8	4.7	1.6	1.0	5.5	1446
Women	64.4	18.6	6.1	3.6	1.4	5.8	1627
Age (years)							
18–24	59.8	19.3	9.2	5.4	1.8	4.5	292
25–34	69.3	16.0	4.4	2.8	2.3	5.2	485
35–44	72.2	14.5	5.6	1.4	0.5	5.8	599
45–54	73.4	13.9	4.5	2.1	0.6	5.6	491
55–64	72.4	14.4	4.1	2.3	1.3	5.4	479
65 years and older	63.2	19.9	6.2	3.2	1.1	6.3	727
Place of residence							
Village	73.3	13.7	4.8	2.4	1.2	4.5	1262
City to 19,999	69.1	16.4	5.8	3.6	0.7	4.4	399
20,000–99,999	68.3	17.3	3.8	2.3	1.4	6.8	672
100,000–499,999	62.7	19.1	7.6	2.8	1.6	6.2	442
500,000 or more residents	57.8	21.4	8.4	3.2	0.8	8.4	299
Education							
Elementary/middle school	64.9	15.6	6.0	4.3	2.5	6.6	467
Basic vocational	71.1	15.6	5.6	1.9	1.6	4.3	712
Medium	68.3	16.5	5.6	3.2	0.8	5.5	1093
Higher	69.1	17.3	4.9	1.8	0.6	6.4	800
Social and professional group							
Staff of managers, especially with higher education	73.5	13.5	5.9	1.5	1.2	4.2	367
Medium staff, technicians	71.6	17.7	4.7	2.4	1.2	2.5	148
Admin office employees	68.0	18.9	5.4	2.6	—	5.0	219
Service workers	69.5	16.1	3.8	2.9	1.1	6.6	255
Skilled workers	80.8	8.1	2.9	1.6	0.9	5.7	351
Unskilled laborers	73.2	10.6	10.7	1.3	0.7	3.4	119
Farmers	75.4	14.6	3.0	0.7	—	6.3	134
Self-employed	80.2	11.0	0.7	1.8	1.1	5.3	132
Unemployed	66.0	16.9	5.6	3.3	2.8	5.5	52
Retirees	65.0	19.3	5.6	3.2	1.0	5.9	798
Pensioners	55.1	21.5	10.8	4.0	2.6	5.9	127
Pupils and students	52.6	19.8	12.2	6.8	1.1	7.4	154
Homemakers and others	57.8	22.8	4.7	3.1	3.6	8.0	217
S/he works at:							
Institute of state, public	73.2	13.7	5.7	1.6	1.0	4.7	400
A partnership of private owners and the state	77.2	10.4	4.4	2.2	—	5.9	357
Private sector, nonfarm	72.1	14.7	4.8	2.7	1.2	4.6	843
Private farming	74.6	15.8	2.9	0.7	—	6.1	140
Per capita income							
Up to PLN 1499	61.6	20.6	6.4	4.3	2.8	4.3	489
From PLN 1500 to PLN 999	69.6	15.7	6.0	6.1	0.6	2.0	418
From PLN 2000 to PLN 2999	71.1	17.7	4.9	1.6	1.1	3.5	557
From PLN 3000 to PLN 3999	67.1	18.8	8.1	3.0	—	3.1	302
PLN 4000 and above	70.9	16.7	4.7	2.2	1.3	4.2	320
Hard to say	70.0	13.3	5.5	1.5	1.7	8.0	412
Refusal to answer	70.3	12.7	3.9	.9	0.5	11.7	575
Evaluation of own material situation							
Bad	44.2	26.3	8.4	8.7	6.9	5.5	227
Medium	61.8	19.2	7.3	2.8	1.2	7.8	1258
Good	77.6	12.7	3.6	1.8	0.4	3.9	1588
Participation in religious practices							
Several times a week	65.0	16.2	7.1	6.0	—	5.7	147
Once a week	73.7	15.4	3.9	1.9	1.0	4.0	1120
1–2 times a month	72.3	15.4	4.7	3.0	0.9	3.8	396
Several times a year	66.9	19.1	6.6	1.9	0.9	4.6	679
Does not participate at all	63.5	17.2	7.4	4.3	2.2	5.4	648
Political views							
Left	64.7	18.5	8.2	4.4	0.9	3.3	647
Center	71.2	16.9	4.6	1.8	1.4	4.3	800
Right	72.3	16.5	4.1	2.2	0.9	3.9	1046
Hard to say	66.9	14.0	6.4	3.2	2.2	7.4	463

**Table 9:** PHQ-8 depression symptoms in Polish population distribution, item 1/8 (*N* = 3073).

Sociodemographic variables	How often have the following problems bothered you in the past 2 weeks? Feeling little interest in the activities you are doing or experiencing little pleasure in doing them					Number of people
	They did not tease at all or for 1 day	For several days	For more than half of the days	Almost every day	Refusal to answer	
	%	%	%	%	%	
Total	67.9	17.5	4.6	6.1	3.9	3073
Gender						
Men	70.6	16.1	4.1	5.3	4.0	1446
Women	65.4	18.7	5.1	6.9	3.9	1627
Age (years)						
18–24	57.1	20.6	10.8	7.5	4.1	292
25–34	63.6	20.2	3.9	8.1	4.3	485
35–44	69.1	16.3	3.7	6.8	4.1	599
45–54	70.1	17.5	3.8	4.8	3.8	491
55–64	71.1	17.7	3.6	4.9	2.7	479
65 years and older	70.3	15.3	4.5	5.5	4.3	727
Place of residence						
Village	69.4	17.5	4.5	5.3	3.3	1262
City to 19,999	70.2	15.4	4.1	7.1	3.1	399
20,000–99,999	70.2	16.7	3.9	4.8	4.4	672
100,000–499,999	65.0	19.2	5.2	7.0	3.6	442
500,000 or more residents	56.9	19.8	6.4	10.2	6.7	299
Education						
Elementary/middle school	67.1	17.2	5.0	6.6	4.1	467
Basic vocational	70.1	17.5	3.3	6.1	3.1	712
Medium	67.5	16.9	5.1	6.5	4.0	1093
Higher	66.8	18.5	4.9	5.5	4.3	800
Social and professional group						
Staff of managers, especially with higher education	68.1	16.3	6.1	6.8	2.6	367
Medium staff, technicians	71.3	14.5	7.7	4.1	2.5	148
Admin office employees	64.2	23.6	4.7	5.1	2.4	219
Service workers	69.5	16.4	4.3	5.0	4.7	255
Skilled workers	73.3	15.6	2.2	4.9	4.0	351
Unskilled laborers	68.0	19.6	4.5	4.6	3.4	119
Farmers	70.6	14.1	4.9	5.4	5.1	134
Self-employed	74.5	13.8	1.8	5.1	4.7	132
Unemployed	64.1	19.0	—	12.3	4.7	52
Retirees	69.9	16.5	4.4	5.3	3.8	798
Pensioners	61.4	20.0	5.5	8.7	4.5	127
Pupils and students	47.3	23.6	13.7	7.9	7.4	154
Homemakers and others	63.6	20.4	0.8	11.2	4.0	217
S/he works at:						
Institute of state, public	69.2	17.6	6.0	4.4	2.7	400
A partnership of private owners and the state	74.4	14.7	2.2	4.4	4.2	357
Private sector, nonfarm	66.7	18.1	5.3	6.5	3.5	843
Private farming	69.6	14.9	4.1	6.5	4.9	140
Per capita income						
Up to PLN 1499	62.5	19.0	6.2	9.5	2.7	489
From PLN 1500 to PLN 999	67.3	18.7	5.5	7.3	1.1	418
From PLN 2000 to PLN 2999	69.0	19.6	4.4	4.7	2.2	557
From PLN 3000 to PLN 3999	66.6	20.4	5.4	5.2	2.5	302
PLN 4000 and above	68.6	16.5	5.4	6.5	3.0	320
Hard to say	70.8	14.7	2.6	6.1	5.9	412
Refusal to answer	69.8	14.4	3.4	4.1	8.2	575
Evaluation of own material situation						
Bad	45.4	26.0	9.0	16.3	3.2	227
Medium	63.2	18.5	5.2	7.9	5.2	1258
Good	74.7	15.5	3.6	3.3	2.9	1588
Participation in religious practices						
Several times a week	70.3	9.5	6.9	8.7	4.6	147
Once a week	73.0	15.7	3.9	4.8	2.6	1120
1–2 times a month	68.2	16.6	6.0	6.5	2.7	396
Several times a year	65.7	21.2	4.4	5.6	3.2	679
Does not participate at all	63.9	20.3	5.1	7.9	2.8	648
Political views						
Left	64.6	20.2	5.7	8.1	1.5	647
Center	69.2	17.2	4.6	5.6	3.3	800
Right	70.5	17.6	4.7	4.4	2.8	1046
Hard to say	68.9	16.0	3.2	7.8	4.1	463

**Table 10:** PHQ-8 depression symptoms in Polish population distribution, item 2/8 (*N* = 3073).

Sociodemographic variables	How often have the following problems bothered you in the past 2 weeks? Feelings of sadness, depression, or hopelessness					Number of people
	They did not tease at all or for 1 day	For several days	For more than half of the days	Almost every day	Refusal to answer	
	%	%	%	%	%	
Total	65.8	21.6	5.0	5.6	2.0	3073
Gender						
Men	70.6	18.9	4.2	4.4	1.9	1446
Women	61.5	24.0	5.7	6.6	2.2	1627
Age						
18–24 years	55.7	26.9	7.5	7.8	2.0	292
25–34	66.2	21.5	4.3	6.0	1.9	485
35–44	73.2	17.1	3.8	3.5	2.3	599
45–54	68.8	21.1	3.7	4.0	2.4	491
55–64	65.9	22.0	4.8	5.2	2.1	479
65 years and older	61.3	23.3	6.4	7.3	1.6	727
Place of residence						
Village	68.9	20.8	3.9	5.1	1.2	1262
City to 19,999	67.1	20.6	4.5	6.7	1.1	399
20,000–99,999	66.7	19.3	6.0	4.8	3.3	672
100,000–499,999	61.6	24.3	6.5	5.9	1.7	442
500,000 or more residents	54.9	27.8	5.8	7.2	4.4	299
Education						
Elementary/middle school	60.2	24.3	7.4	6.7	1.5	467
Basic vocational	67.8	21.5	3.9	5.8	1.0	712
Medium	65.6	20.6	5.3	6.1	2.4	1093
Higher	67.6	21.5	4.1	3.9	2.8	800
Social and professional group						
Staff of managers, especially with higher education	70.6	19.0	5.8	3.1	1.5	367
Medium staff, technicians	70.7	18.9	3.9	5.7	0.8	148
Admin office employees	65.6	23.2	5.0	3.4	2.7	219
Service workers	64.9	25.6	2.5	4.6	2.4	255
Skilled workers	75.7	17.6	1.7	3.8	1.3	351
Unskilled laborers	66.4	20.7	7.6	4.3	1.1	119
Farmers	76.2	14.0	5.6	3.2	1.0	134
Self-employed	77.6	15.1	1.7	3.9	1.7	132
Unemployed	50.7	35.9	2.4	8.7	2.3	52
Retirees	62.0	23.5	5.7	7.2	1.6	798
Pensioners	51.6	24.0	11.1	9.8	3.5	127
Pupils and students	52.1	25.8	7.4	10.0	4.7	154
Homemakers and others	61.0	22.5	5.6	6.6	4.3	217
S/he works at:						
Institute of state, public	70.9	20.0	4.6	2.7	1.8	400
A partnership of private owners and the state	72.5	20.4	1.8	3.8	1.5	357
Private sector, nonfarm	68.0	19.6	5.3	4.9	2.2	843
Private farming	77.7	12.9	5.3	3.1	1.0	140
Per capita income						
Up to PLN 1499	57.7	24.1	6.9	9.7	1.6	489
From PLN 1500 to PLN 999	66.2	21.5	5.9	6.5	—	418
From PLN 2000 to PLN 2999	66.7	24.2	4.8	3.7	0.7	557
From PLN 3000 to PLN 3999	65.5	22.6	5.9	5.3	0.7	302
PLN 4000 and above	67.2	20.5	5.3	6.0	1.0	320
Hard to say	67.6	20.4	4.2	5.2	2.6	412
Refusal to answer	69.6	18.1	3.0	3.4	6.0	575
Evaluation of own material situation						
Bad	40.5	31.6	11.1	15.6	1.1	227
Medium	57.8	26.5	5.8	7.1	2.8	1258
Good	75.7	16.3	3.5	2.9	1.5	1588
Participation in religious practices						
Several times a week	63.8	22.5	7.2	6.1	0.4	147
Once a week	69.7	20.1	4.1	5.1	1.1	1120
1–2 times a month	67.2	23.2	4.9	4.5	0.1	396
Several times a year	67.1	20.9	5.6	4.7	1.7	679
Does not participate at all	59.9	24.2	6.0	8.1	1.8	648
Political views						
Left	60.3	25.4	6.1	7.3	0.9	647
Center	67.0	21.8	5.0	4.9	1.4	800
Right	70.0	20.6	4.4	3.9	1.1	1046
Hard to say	65.7	18.9	5.8	7.7	1.9	463

**Table 11:** PHQ-8 depression symptoms in Polish population distribution, item 3/8 (*N* = 3073).

Sociodemographic variables	How often have the following problems bothered you in the past 2 weeks? Trouble sleeping or intermittent sleep or sleeping too much					Number of people
	They did not tease at all or for 1 day	For several days	For more than half of the days	Almost every day	Refusal to answer	
	%	%	%	%	%	
Total	60.0	20.3	6.8	11.4	1.6	3073
Gender						
Men	65.4	18.9	5.9	8.3	1.5	1446
Women	55.3	21.5	7.5	14.1	1.6	1627
Age						
18–24 years	59.6	20.0	6.5	11.6	2.3	292
25–34	63.3	18.2	6.0	10.9	1.5	485
35–44	64.4	20.2	4.1	9.5	1.8	599
45–54	64.6	19.9	7.2	6.8	1.5	491
55–64	61.1	19.4	6.8	10.8	2.0	479
65 years and older	5.6	22.6	9.2	16.7	0.8	727
Place of residence						
Village	62.8	20.6	5.1	10.1	1.3	1262
City to 19,999	63.3	19.3	5.3	11.4	0.7	399
20,000–99,999	58.9	18.7	7.7	12.4	2.3	672
100,000–499,999	56.4	19.6	9.4	13.4	1.2	442
500,000 or more residents	51.6	24.6	9.5	11.6	2.7	299
Education						
Elementary/middle school	56.7	19.8	6.7	15.8	1.0	467
Basic vocational	62.8	19.6	6.0	11.1	0.5	712
Medium	59.6	20.5	7.2	10.6	2.2	1093
Higher	60.0	20.9	6.9	10.3	1.9	800
Social and professional group						
Staff of managers, especially with higher education	61.0	21.4	6.8	9.2	1.6	367
Medium staff, technicians	62.7	19.1	8.3	9.9	—	148
Admin office employees	62.2	17.9	7.2	10.2	2.4	219
Service workers	56.2	26.1	5.9	10.1	1.7	255
Skilled workers	70.5	18.4	3.4	6.1	1.7	351
Unskilled laborers	67.7	15.1	7.3	8.9	0.9	119
Farmers	68.4	18.6	7.1	5.0	1.0	134
Self-employed	72.3	18.5	1.8	5.7	1.7	132
Unemployed	67.2	11.7	4.2	15.5	1.5	52
Retirees	54.1	21.2	8.2	15.5	0.9	798
Pensioners	52.4	19.8	10.1	15.7	2.0	127
Pupils and students	55.9	22.4	6.9	11.1	3.7	154
Homemakers and others	52.2	20.2	7.4	17.6	2.5	217
S/he works at:						
Institute of state, public	65.9	17.9	6.2	8.1	1.9	400
A partnership of private owners and the state	64.8	19.6	4.9	8.7	1.9	357
Private sector, nonfarm	62.7	21.5	5.6	8.7	1.5	843
Private farming	67.6	20.2	6.0	5.3	1.0	140
Per capita income						
Up to PLN 1499	53.0	21.1	8.3	16.6	1.0	489
From PLN 1500 to PLN 999	58.9	20.6	7.4	12.9	0.2	418
From PLN 2000 to PLN 2999	59.2	21.8	8.0	10.9	.0.1	557
From PLN 3000 to PLN 3999	56.7	23.2	6.6	13.3	0.2	302
PLN 4000 and above	59.4	23.4	7.2	8.9	1.2	320
Hard to say	65.4	15.8	5.8	11.0	2.0	412
Refusal to answer	65.9	17.9	4.2	7.1	4.8	575
Evaluation of own material situation						
Bad	39.8	27.4	8.7	23.6	0.6	227
Medium	53.8	22.4	9.4	12.6	1.8	1258
Good	67.9	17.6	4,.4	8.7	1.4	1588
Participation in religious practices						
Several times a week	53.8	18.8	8.0	18.3	1.2	147
Once a week	62.5	21.1	6.4	9.4	.0.6	1120
1–2 times a month	61.4	22.2	5.2	10.6	0.6	396
Several times a year	60.1	18.9	8.0	12.1	1.0	679
Does not participate at all	57.6	21.0	6.5	13.2	1.7	648
Political views						
Left	54.6	24.6	8.9	11.4	0.5	647
Center	63.6	19.0	7.0	9.4	1.1	800
Right	61.9	20.1	6.0	11.2	0.8	1046
Hard to say	59.8	17.6	5.5	15.7	1.4	463

**Table 12:** PHQ-8 depression symptoms in Polish population distribution, item 4/8 (*N* = 3073).

Sociodemographic variables	How often have the following problems bothered you in the past 2 weeks? Feelings of fatigue or lack of energy					Number of people
	They did not tease at all or for 1 day	For several days	For more than half of the days	Almost every day	Refusal to answer	
	%	%	%	%	%	
Total	45.5	33.8	8.8	10.3	1.6	3073
Gender						
Men	50.4	31.7	8.8	7.5	1.4	1446
Women	41.2	35.6	8.8	12.7	1.7	1627
Age (years)						
18–24 years	43.2	28.5	14.4	12.0	1.9	292
25–34	45.4	31.3	9.3	12.7	1.3	485
35–44	47.8	32.7	7.9	9.3	2.2	599
45–54	44.4	38.2	7.8	8.0	1.6	491
55–64	48.6	34.7	5.2	10.0	1.5	479
65 years and older	43.5	34.9	10.0	10.5	1.1	727
Place of residence						
Village	47.6	35.1	6.6	9.7	1.0	1262
City to 19,999	46.8	31.7	9.4	11.9	0.3	399
20,000–99,999	47.8	31.3	9.2	9,2	2,6	672
100,000–499,999	43.1	31.5	11.8	12.6	1,0	442
500,000 or more residents	34.0	40.2	11.8	9.7	4,3	299
Education						
Elementary/middle school	39.6	36.6	11.2	11.8	0.7	467
Basic vocational	50.5	30.6	7.6	10.8	.0.6	712
Medium	46.1	34.0	8.4	9.5	2.0	1093
Higher	43.8	34.8	9.0	10.0	2.4	800
Social and professional group						
Staff of managers, especially with higher education	44.2	34.1	9.4	11.1	1.2	367
Medium staff, technicians	45.3	31.9	12.3	10.5	—	148
Admin office employees	39,3	37,7	8.0	12.2	2.9	219
Service workers	44.8	38.0	7.8	8.0	1.5	255
Skilled workers	53.5	33.9	5.2	6.3	1.1	351
Unskilled laborers	38.1	41.9	12.4	7.6	—	119
Farmers	54.3	30.1	6.2	8.4	1.0	134
Self-employed	59.0	29.7	3.9	5.7	1.7	132
Unemployed	46.2	29.7	7.0	14.8	2.3	52
Retirees	45.3	34.1	9.0	10.3	1.2	798
Pensioners	36.7	31.2	12.1	17.1	2.9	127
Pupils and students	37.5	31.3	13.9	12.7	4.6	154
Homemakers and others	44.5	28.9	9.9	14.3	2.4	217
S/he works at:						
Institute of state, public	45.7	35.0	9.3	8.7	1.3	400
A partnership of private owners and the state	49.1	35.4	7.0	7.2	1.3	357
Private sector, nonfarm	44.3	35.7	8.3	10.1	1.7	843
Private farming	54.7	29.6	4.5	10.2	1.0	140
Per capita income						
Up to PLN 1499	40.0	33.8	9.8	15.0	1.3	489
From PLN 1500 to PLN 999	42.0	37.0	9.6	11.4	—	418
From PLN 2000 to PLN 2999	47.0	35.5	8.8	8.5	0.3	557
From PLN 3000 to PLN 3999	40.2	39.5	5.8	13.7	0.8	302
PLN 4000 and above	49.2	31.7	7.9	10.6	0.6	320
Hard to say	52.2	28.5	9.3	8.6	1.4	412
Refusal to answer	47.5	31.8	9.1	6.4	5.2	575
Evaluation of own material situation						
Bad	29.6	29.6	15.8	23.7	1.3	227
Medium	38.5	36.6	10.8	11.9	2.2	1258
Good	53.5	32.2	6.2	7.1	1.1	1588
Participation in religious practices						
Several times a week	42.7	32.3	7.4	16.5	1.2	147
Once a week	48.2	36.0	7.1	8.0	0.7	1120
1–2 times a month	43.4	35,9	10.4	10.3	—	396
Several times a year	43.8	35.1	10.7	9.4	1.1	679
Does not participate at all	45.7	28.9	9.7	14.3	1.3	648
Political views						
Left	43.8	30.7	10.3	14.8	0.4	647
Center	45.4	37.1	9.4	7.2	0.9	800
Right	47.1	36.0	7.5	8.4	1.0	1046
Hard to say	47.4	28.2	9.8	13.1	1.6	463

**Table 13:** PHQ-8 depression symptoms in Polish population distribution, item 5/8 (*N* = 3073).

Sociodemographic variables	How often have the following problems bothered you in the past 2 weeks? Lack of appetite or overeating					Number of people
	They did not tease at all or for 1 day	For several days	For more than half of the days	Almost every day	Refusal to answer	
	%	%	%	%	%	
Total	83.1	9.4	3.2	2.9	1.5	3073
Gender						
Men	85.2	8.4	3.0	2.0	1.4	1446
Women	81.2	10.2	3.4	3.6	1.6	1627
Age (years)						
18–24	75.9	12.2	4.8	5.2	1.9	292
25–34	80.2	10.1	3.8	4.3	1.6	485
35–44	84.7	9.5	2.7	1.5	1.6	599
45–54	85.1	8.6	3.5	1.3	1.4	491
55–64	84.8	8.9	1.8	2.6	1.9	479
65 years and older	84.0	8.4	3.4	3.3	0.9	727
Place of residence						
Village	84.8	8.6	2.5	3.3	0.9	1262
City to 19,999	82.6	9.1	4.5	3.3	0.5	399
20,000–99,999	83.5	8.6	3.0	2.4	2.5	672
100,000–499,999	81.8	11.1	3.3	2.7	1.0	442
500,000 or more residents	77.6	11.9	5.1	1.9	3.6	299
Education						
Elementary/middle school	79.4	12.2	3.8	4.1	0.5	467
Basic vocational	84.9	9.0	3.0	2.4	0.7	712
Medium	83.3	8.5	3.1	3.1	2.0	1093
Higher	83.3	9.2	3.2	2.2	2.0	800
Social and professional group						
Staff of managers, especially with higher education	82.6	10.1	3.6	2.5	1.2	367
Medium staff, technicians	83.7	9.1	5.2	2.1	—	148
Admin office employees	84.7	8.9	3.0	2.0	1.4	219
Service workers	85.1	7.8	2.6	3.4	1.2	255
Skilled workers	85.9	9.0	2.0	1.4	1.7	351
Unskilled laborers	79.1	13.6	4.8	1.6	0.9	119
Farmers	88.2	8.9	1.9	—	1.0	134
Self-employed	85.0	7.7	1.1	4.5	1.7	132
Unemployed	80.3	11.4	—	6.7	1.6	52
Retirees	85.0	7.7	3.2	2.8	1.2	798
Pensioners	76.5	9.0	4.7	7.8	2.0	127
Pupils and students	70.3	15.0	6.6	4.2	3.9	154
Homemakers and others	79.2	12.0	2.7	3.7	2.4	217
S/he works at:						
Institute of state, public	84.2	9.9	3.4	1.3	1.2	400
A partnership of private owners and the state	85.8	8.1	2.4	1.8	1.9	357
Private sector, nonfarm	81.9	9.4	4.1	3.3	1.4	843
Private farming	87.9	10.2	1.0	—	1.0	140
Per capita income						
Up to PLN 1499	81.4	9.7	3.0	4.9	1.0	489
From PLN 1500 to PLN 999	81.9	10.2	4.4	3.3	0.2	418
From PLN 2000 to PLN 2999	86.2	8.6	3.1	1.9	.0.2	557
From PLN 3000 to PLN 3999	82.6	9.4	5.1	2.3	0.5	302
PLN 4000 and above	84.0	10.1	2.6	2.7	0.6	320
Hard to say	83.1	9.2	2.9	3.4	1.3	412
Refusal to answer	82.0	8.9	2.2	1.9	5.0	575
Evaluation of own material situation						
Bad	70.2	14.7	5.5	8.2	1.3	227
Medium	81.3	10.1	3.4	3.3	1.8	1258
Good	86.3	8.0	2.7	1.8	1.2	1588
Participation in religious practices						
Several times a week	82.4	7.6	5.9	3.8	0.4	147
Once a week	87.1	7.6	2.6	2.1	0.6	1120
1–2 times a month	83.4	11,2	3,3	1.5	0.6	396
Several times a year	82.4	10.4	3.3	2.8	1.1	679
Does not participate at all	79.0	10.7	3.9	5.3	1.2	648
Political views						
Left	80.0	9.6	5.2	4.6	0.5	647
Center	85.4	9.4	2.0	2.4	0.8	800
Right	84.7	8.9	3.5	2.0	0.9	1046
Hard to say	82.6	10.6	2.5	3.5	0.8	463

**Table 14:** PHQ-8 depression symptoms in Polish population distribution, item 6/8 (*N* = 3073).

Sociodemographic variables	How often have the following problems bothered you in the past 2 weeks? Feeling dissatisfied with yourself—or feeling that you suck or that you have let yourself or your family down					Number of people
	They did not tease at all or for 1 day	For several days	For more than half of the days	Almost every day	Refusal to answer	
	%	%	%	%	%	
Total	79.5	11.9	2.8	3.5	2.3	3073
Gender						
Men	82.2	10.8	2.6	2,4	2.0	1446
Women	77.0	12.8	3.1	4.6	2.6	1627
Age (years)						
18–24	67.7	15.9	5.6	8.9	1.9	292
25–34	77.6	12.1	2.4	5.6	2.2	485
35–44	82.3	11.3	1.8	1.9	2.6	599
45–54	79.9	12.2	2.3	3.1	2.5	491
55–64	81.6	11.1	2.7	1.9	2.6	479
65 years and older	81.4	10.8	3.2	2.7	2.0	727
Place of residence						
Village	82.1	11.0	1.7	3.4	1.8	1262
City to 19,999	83.3	9.8	2.3	3.8	0.9	399
20,000–99,999	78.2	12.0	3.7	2.6	3.5	672
100,000–499,999	76.5	12.6	4.2	4.8	1.9	442
500,000 or more residents	70.4	16.9	4.6	3.8	4.3	299
Education						
Elementary/middle school	78.2	12.0	4.4	4.0	1.5	467
Basic vocational	81.6	10.6	2.4	3.1	2.2	712
Medium	79.1	11.9	2.8	4.1	2.2	1093
Higher	78.8	12.9	2.4	2.9	3.0	800
Social and professional group						
Staff of managers, especially with higher education	82.0	10.0	3.6	2.5	2.0	367
Medium staff, technicians	80.9	13.4	1.5	4.3	—	148
Admin office employees	75.6	15.7	3.0	3.9	1.8	219
Service workers	81.5	10.0	2.5	4.2	1.7	255
Skilled workers	86.5	6.7	2.5	1.9	2.4	351
Unskilled laborers	75.2	17.8	1.5	3.4	2.1	119
Farmers	85.5	11.2	1.3	—	2.0	134
Self-employed	87.4	6.5	—	3.1	3.0	132
Unemployed	69.5	15.6	5.7	6.1	3.1	52
Retirees	81.4	11.3	3.0	2.4	2.0	798
Pensioners	68.9	16.0	6.6	5.0	3.5	127
Pupils and students	59.2	19.5	4.8	11.8	4.6	154
Homemakers and others	74.0	14.7	1.7	5.5	4.1	217
S/he works at:						
Institute of state, public	79.8	11.6	2.8	3.5	2.2	400
A partnership of private owners and the state	82.1	9.4	3.9	2.6	1.9	357
Private sector, nonfarm	80.8	11.5	1.7	3.9	2.1	843
Private farming	87.1	9.8	1.2	—	1.9	140
Per capita income						
Up to PLN 1499	75.8	14.4	3.3	5.0	1.4	489
From PLN 1500 to PLN 999	80.4	12.5	2.7	4.0	0.4	418
From PLN 2000 to PLN 2999	82.1	12.0	2.0	2.8	1.1	557
From PLN 3000 to PLN 3999	80.5	12.7	3.9	1.7	1.2	302
PLN 4000 and above	77.7	14.3	2.5	4.9	0.7	320
Hard to say	79.2	10.3	3.7	4.7	2.1	412
Refusal to answer	80.1	8.4	2.3	2.0	7.1	575
Evaluation of own material situation						
Bad	65.0	14.8	6.1	12.8	1.3	227
Medium	75.7	13.4	3.3	4.1	3.5	1258
Good	84.6	10.2	2.0	1.8	1.5	1588
Participation in religious practices						
Several times a week	80.6	10.5	4.6	2.3	2.0	147
Once a week	83.2	11.4	2.2	2.2	1.1	1120
1–2 times a month	82.3	11.9	2.1	2.8	0.9	396
Several times a year	78.8	14.4	2.9	2.4	1.5	679
Does not participate at all	75.0	11.0	4.1	7.8	2.1	648
Political views						
Left	76.9	12.2	4.3	5.2	1.3	647
Center	82.6	11,0	2.7	3.0	0.8	800
Right	81.3	12.7	2.2	2.2	1.5	1046
Hard to say	77.7	11.3	2.7	5.5	2.8	463

**Table 15:** PHQ-8 depression symptoms in Polish population distribution, item 7/8 (*N* = 3073).

Sociodemographic variables	How often have the following problems bothered you in the past 2 weeks? Problems concentrating (e.g., when reading a newspaper or watching TV)					Number of people
	They did not tease at all or for 1 day	For several days	For more than half of the days	Almost every day	Refusal to answer	
	%	%	%	%	%	
Total	79.0	12.9	3.5	3.0	1.6	3073
Gender						
Men	81.7	11.6	2.9	2.5	1.4	1446
Women	76.6	14.1	4.1	3.5	1.7	1627
Age (years)						
18–24	68.2	17.2	7.2	5.1	2,3	292
25–34	74.7	17.3	3.1	3.8	1.1	485
35–44	82.0	11.4	2.6	2.1	1.8	599
45–54	82.1	10.5	3.5	2.4	1.5	491
55–64	84.0	10.1	2.1	2.3	1.6	479
65 years and older	78.4	12.9	4.1	3.2	1.4	727
Place of residence						
Village	82.4	11.1	3.3	2.3	0.9	1262
City to 19,999	84.3	9.2	3.9	2.3	0.3	399
20,000–99,999	78.6	13.1	2.8	2.6	2.9	672
100,000–499,999	72.3	16.4	4.2	5.7	1.4	442
500,000 or more residents	68.3	19.8	4.8	3.9	3.3	299
Education						
Elementary/middle school	75.0	15.2	5.1	3.7	1.0	467
Basic vocational	83.1	10.3	2.8	3.2	0.6	712
Medium	79.7	11.5	3.7	3.1	2.0	1093
Higher	76.7	15.8	3.0	2.2	2.2	800
Social and professional group						
Staff of managers, especially with higher education	75.4	17.0	4.1	2.5	1.0	367
Medium staff, technicians	79.7	15.2	3.3	1.8	—	148
Admin office employees	77.7	16.3	1.9	2.5	1.6	219
Service workers	77.5	14.1	3,7	4.0	0.7	255
Skilled workers	86.9	7.6	1.6	1.7	2.2	351
Unskilled laborers	82.0	10.0	4.1	2.9	0.9	119
Farmers	90.8	6.6	1.6	—	1.0	134
Self-employed	86.5	10.2	0.5	1.1	1.7	132
Unemployed	86,8	3,8	1.1	6.8	1.6	52
Retirees	78.8	12.5	3.9	3.5	1.4	798
Pensioners	74.8	13.0	5.6	4.5	2.0	127
Pupils and students	57.6	22.9	9.5	5.3	4.6	154
Homemakers and others	77.9	11.8	4.0	4.0	2.4	217
S/he works at:						
Institute of state, public	80.4	11.9	4.3	1.9	1.4	400
A partnership of private owners and the state	76.7	17.5	2.3	1.7	1.8	357
Private sector, nonfarm	80.4	12.1	2,7	3.5	1.2	843
Private farming	90.0	7.4	1.5	—	1.0	140
Per capita income						
Up to PLN 1499	79.2	11.7	4.5	3.6	1.0	489
From PLN 1500 to PLN 999	76.9	14.7	3.9	4.2	0.3	418
From PLN 2000 to PLN 2999	80.2	13.3	3.6	2.6	0.4	557
From PLN 3000 to PLN 3999	78.2	15.2	2.6	3.8	0.2	302
PLN 4000 and above	77.4	16.2	3.9	1.7	.8	320
Hard to say	82.5	10.2	3.0	2.8	1.5	412
Refusal to answer	78.2	11.1	3.1	2.4	5.2	575
Evaluation of own material situation						
Bad	68.1	13.5	4.8	12.3	1.3	227
Medium	76.3	14.1	4.7	2.8	2.0	1258
Good	82.7	11.9	2.4	1.8	1.2	1588
Participation in religious practices						
Several times a week	79.2	9.4	7.0	3.2	1.2	147
Once a week	83.5	10.8	2.2	2.8	0.7	1120
1–2 times a month	79.2	15.0	3.8	1.8	0.3	396
Several times a year	79.2	13.1	4.5	2.1	1.1	679
Does not participate at all	73.5	16.2	4.2	4.7	1.4	648
Political views						
Left	73.0	16.6	5.7	4.1	0.6	647
Center	81.6	13.0	2.3	2.5	0.7	800
Right	82.1	11.8	3.2	1.9	1.0	1046
Hard to say	78.9	11.3	3.4	5.1	1.3	463

**Table 16:** PHQ-8 depression symptoms in Polish population distribution, item 8/8 (*N* = 3073).

Sociodemographic variables	How often have the following problems bothered you in the past 2 weeks? Moving or speaking so slowly that others could notice or, on the contrary, inability to sit still, moving much more					Number of people
	They did not tease at all or for 1 day	For several days	For more than half of the days	Almost every day	Refusal to answer	
	%	%	%	%	%	
Total	86.1	6.9	2.0	2.5	2.3	3073
Gender						
Men	87.2	6.5	1.7	2.7	1.9	1446
Women	85.2	7.4	2.3	2.4	2.7	1627
Age (years)						
18–24	83.0	9.0	3.1	1.9	3.0	292
25–34	88.5	5.5	1.7	2.4	1.9	485
35–44	90.7	4.9	1.0	1.3	2.1	599
45–54	90.5	5.1	1.4	1.0	1.9	491
55–64	86.6	5.4	1.9	2.4	3.6	479
65 years and older	78.8	11.0	3.2	4.9	2.0	727
Place of residence						
Village	87.6	6.1	2.0	2.6	1.6	1262
City to 19,999	87.0	7.9	1.5	2.6	0.9	399
20,000–99, 999	84.4	6.6	2.7	2.7	3.6	672
100,000–499, 999	87.6	6.5	1.1	2.4	2.4	442
500,000 or more residents	80.6	10.5	2.5	2.1	4.2	299
Education						
Elementary/middle school	80.0	8.6	3.8	5.2	2.4	467
Basic vocational	87.1	6,5	1.9	3.4	1.2	712
Medium	85.6	7,4	2.3	2.1	2.7	1093
Higher	89.6	5,8	0.8	0.9	2.8	800
Social and professional group						
Staff of managers, especially with higher education	90.3	6.4	1.2	0.4	1.6	367
Medium staff, technicians	93.6	2.1	2.1	2.1	—	148
Admin office employees	94.5	2.9	—	0.2	2.4	219
Service workers	87.8	7.7	1.9	0.9	1.6	255
Skilled workers	91.6	4.7	0.6	0.9	2.2	351
Unskilled laborers	84.3	8.7	1.7	4.3	.9	119
Farmers	91.6	4.1	1.3	.7	2.3	134
Self-employed	91.7	5.9	—	—	2.4	132
Unemployed	84.9	6.2	5.6	1.0	2.3	52
Retirees	80.9	9.9	2.8	4.4	2.1	798
Pensioners	73.0	6.8	4.8	10.0	5.5	127
Pupils and students	79.6	8.9	4.5	1.9	5.2	154
Homemakers and others	81.1	7.4	2.6	4.8	4.1	217
S/he works at:						
Institute of state, public	89.9	6.0	1.1	1.6	1.4	400
A partnership of private owners and the state	91.1	5.3	0.2	0.2	3.1	357
Private sector, nonfarm	90.4	5.2	1.6	1.3	1.5	843
Private farming	91.3	4.6	1.2	.7	2.2	140
Per capita income						
Up to PLN 1499	81.2	11.4	2.1	3.7	1.5	489
From PLN 1500 to PLN 999	84.4	7.7	5.3	1.8	0.8	418
From PLN 2000 to PLN 2999	88.4	6.5	1.5	2.8	0.8	557
From PLN 3000 to PLN 3999	87.4	9.2	1.0	1.5	0.8	302
PLN 4000 and above	91.7	4.3	0.8	1.8	1.4	320
Hard to say	85.9	6.0	1,8	4.2	2.1	412
Refusal to answer	86.1	4.0	1.5	1.5	6.9	575
Evaluation of own material situation						
Bad	74.0	9.1	5.2	8.3	3.4	227
Medium	83.5	8.2	2.5	2.7	3.1	1258
Good	90.0	5.7	1.2	1.6	1.5	1588
Participation in religious practices						
Several times a week	82.9	9.2	3.3	2.3	2.3	147
Once a week	88.3	6.0	1.6	2.9	1.3	1120
1–2 times a month	87.0	7.1	2.6	1.9	1.4	396
Several times a year	86.0	8.7	2.4	1.2	1.7	679
Does not participate at all	86.1	6.6	2.0	3.6	1.7	648
Political views						
Left	86.8	7.7	2.4	1.9	1.0	647
Center	86.9	7.0	2.9	1.9	1.3	800
Right	87.1	7.7	0.9	2.5	1.7	1046
Hard to say	84.1	5.0	2.7	5.1	3.1	463

## Data Availability

No publicly available datasets exist; however, public sector researchers interested in accessing the data will be allowed to agree upon data availability on request through the corresponding author (ptoczyski@aps.edu.pl). Access to data is restricted for private sector, according to confidentiality policy.
